# Supplementary material for: Operating Regimes of Signaling Cycles: Statics, Dynamics, and Noise Filtering
Source: PLoS Comput Biol. 2007 Dec 21;3(12):e246. doi: 10.1371/journal.pcbi.0030246 (PMC2230677; doi:10.1371/journal.pcbi.0030246)
Supplement: Figure S1 — Plots show the output oscillations O of the hyperbolic, signal-transducing, threshold-hyperbolic, and ultrasensitive switches, respectively (normalized by the steady-state saturation value of each cycle), shown in Figure 2, in response to an input of the form . The magnitude of O is color-coded and shown as a function of the input amplitude a and frequency w. Output oscillations increase with increasing a and decrease with increasing w, as expected. The four cycles, however, respond very differently to their inputs. The parameters used for the cycles are the same as those in Figure 2, and nM except for the threshold-hyperbolic switch, where nM. (808 KB PPT) [file pcbi.0030246.sg001.ppt]

## Slide 1
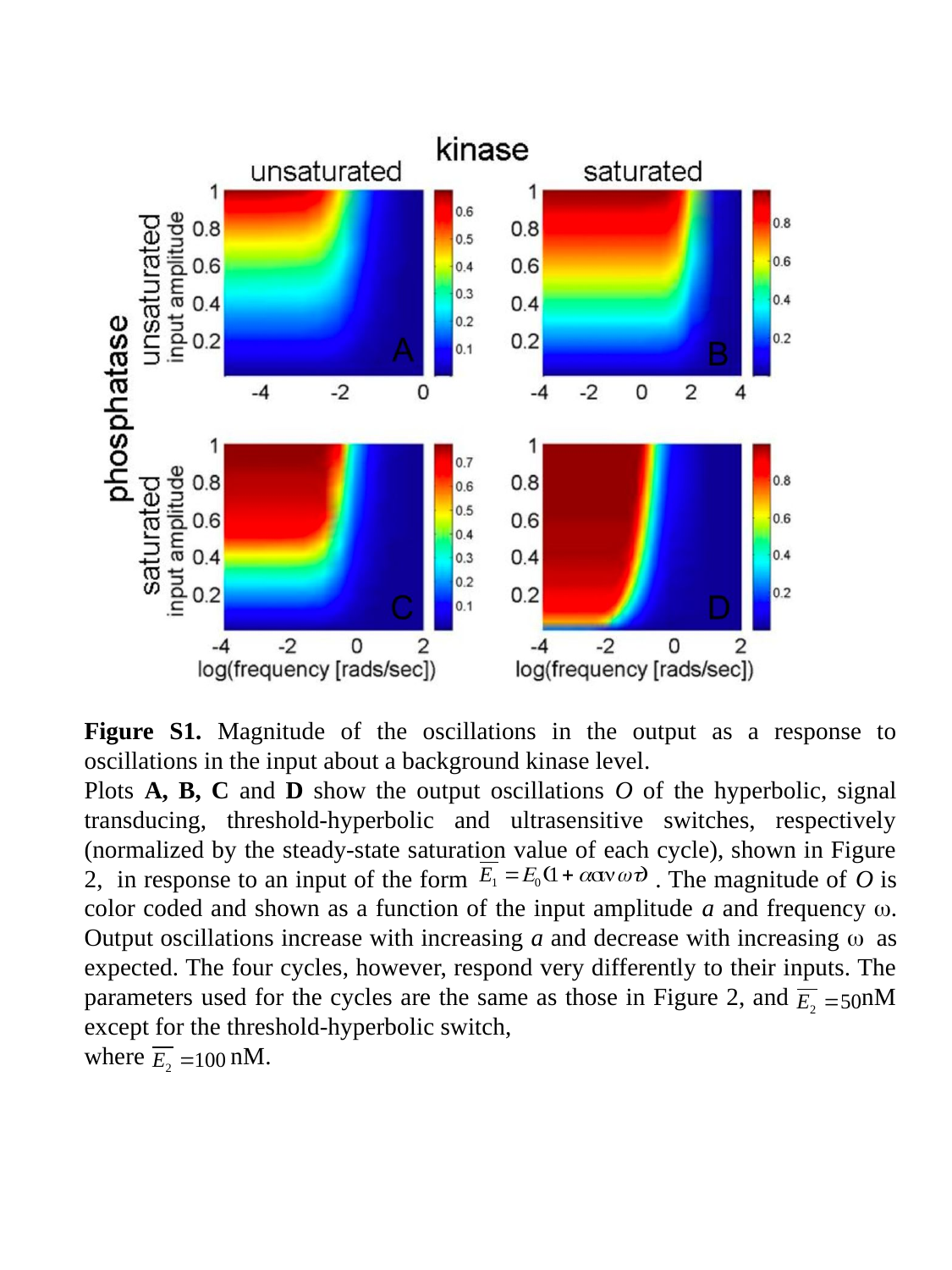

Figure S1. Magnitude of the oscillations in the output as a response to oscillations in the input about a background kinase level.
Plots A, B, C and D show the output oscillations O of the hyperbolic, signal transducing, threshold-hyperbolic and ultrasensitive switches, respectively (normalized by the steady-state saturation value of each cycle), shown in Figure 2, in response to an input of the form . The magnitude of O is color coded and shown as a function of the input amplitude a and frequency . Output oscillations increase with increasing a and decrease with increasing  as expected. The four cycles, however, respond very differently to their inputs. The parameters used for the cycles are the same as those in Figure 2, and nM except for the threshold-hyperbolic switch,
where nM.
