# Supplementary material for: Operating Regimes of Signaling Cycles: Statics, Dynamics, and Noise Filtering
Source: PLoS Comput Biol. 2007 Dec 21;3(12):e246. doi: 10.1371/journal.pcbi.0030246 (PMC2230677; doi:10.1371/journal.pcbi.0030246)
Supplement: Text S1 — (86 KB PDF) [file pcbi.0030246.sd001.pdf]

Text S1

Derivation of Equation 3

We start with the mass action kinetics description of the reactions specified by Equations 1 and 2. There are six chemical species and three conservation relations (the kinase, phosphatase and substrate protein are conserved), yielding a total of three variables. Letting C_1 (C_2) denote the concentration of the inactive (active) enzyme-substrate complex IE_1 (AE_1), we write down the mass action kinetics equations for the enzyme-substrate complexes and for the amount of active protein $\overline{A}$ yields

$$\frac{d\overline{A}}{dt} = k_1 C_1 - k_2 C_2 \tag{5}$$

$$\begin{aligned} \frac{dC_1}{dt} &= a_1\big[(\overline{S} - \overline{A} - C_1)(\overline{E_1} - C_1) - K_1 C_1\big] \\ &= a_1\big[C_1^2 - (K_1 + \overline{E_1} + \overline{S} - \overline{A})C_1 + \overline{E_1}(\overline{S} - \overline{A})\big] \end{aligned} \tag{6}$$

$$\begin{aligned} \frac{dC_2}{dt} &= a_2\big[(\overline{A} - C_2)(\overline{E_2} - C_2) - K_2 C_2\big] \\ &= a_2\big[C_2^2 - (K_2 + \overline{E_2} + \overline{A})C_2 + \overline{E_2}\,\overline{A}\big], \end{aligned} \tag{7}$$

where $\overline{S}$ denotes the total amount of substrate protein, a_1 and a_2 are the association rate constants of the two enzymatic reactions and K_1 and K_2 the Michaelis Menten constants.

To apply the tQSSA, we then hypothesize that the complexes have faster dynamics than the active protein and that they are always at equilibrium with respect to the active substrate protein. This allows us to substitute C_1 and C_2 in Equation 5 with the equilibrium values, which are in turn found by setting the left hand side of Equations 6 and 7 to zero and solving for the complexes.

So doing yields

$$C_1 = \frac{K_1 + \overline{E_1} + \overline{S} - \overline{A}}{2}\big(1 - \sqrt{1 - 4r_1}\big) \quad \text{and} \quad C_2 = \frac{K_2 + \overline{E_2} + \overline{A}}{2}\big(1 - \sqrt{1 - 4r_2}\big)$$

where

$$r_1 = \frac{\overline{E_1}(\overline{S} - \overline{A})}{\big(K_1 + \overline{E_1} + \overline{S} - \overline{A}\big)^2} \quad \text{and} \quad r_2 = \frac{\overline{E_2}\,\overline{A}}{\big(K_2 + \overline{E_2} + \overline{A}\big)^2}.$$

To further simplify the equilibrium expressions for the complexes, we approximate to first order in r_1 and r_2 (which is reasonable when $r_1 \ll 1$ and $r_2 \ll 1$), yielding the following expressions for C_1 and C_2:

$$C_1 = \frac{\overline{E_1}(\overline{S} - \overline{A})}{K_1 + \overline{E_1} + \overline{S} - \overline{A}} \quad \text{and} \quad C_2 = \frac{\overline{E_2}\,\overline{A}}{K_2 + \overline{E_2} + \overline{A}}. \tag{8}$$

These expressions are finally inserted into Equation 5 to yield the signaling cycle equation (Equation 3).

In [32], R. Tzafriri describes in full detail how to obtain the same result for a single enzymatic reaction in a self-consistent manner. In particular, he finds conditions under which the complexes indeed reach equilibrium with respect to the substrate, and under which the first order approximation of the square root is valid. The same argument carries through for each enzymatic reaction in the signaling cycle. In particular, the tQSSA is expected to hold when

1. Either $K_1 + \overline{E_1} \gg \overline{S}$ or $K_1 + \overline{S} - \overline{A} \gg \overline{E_1}$, and
2. Either $K_2 + \overline{E_2} \gg \overline{S}$ or $K_2 + \overline{A} \gg \overline{E_2}$.

As these inequalities are better satisfied, the tQSSA describes the signaling cycle better. Similarly, whereas a Michaelis Menten approximation would be valid only at low enzyme concentrations, an inspection of the conditions above shows the tQSSA is also valid when the enzyme concentrations are high. The conditions we use to define the four signaling regimes of the cycle are consistent with the sufficient conditions for the validity of the tQSSA.
