# Supplementary material for: Operating Regimes of Signaling Cycles: Statics, Dynamics, and Noise Filtering
Source: PLoS Comput Biol. 2007 Dec 21;3(12):e246. doi: 10.1371/journal.pcbi.0030246 (PMC2230677; doi:10.1371/journal.pcbi.0030246)
Supplement: Text S2 — (84 KB PDF) [file pcbi.0030246.sd002.pdf]

Text S2

Equations for four regimes

Regime 1: both kinase and phosphatase are saturated : ultrasensitive

This regime was first identified in [27], where its steady state behavior was analyzed. Equation 3 reduces to

$$\frac{d\overline{A}}{dt} = k_1\overline{E_1} - k_2\overline{E_2},$$

indicating that in this regime the signaling cycle effectively integrates the difference of its (scaled) input and a reference level specified by the (scaled) phosphatase level. When the difference maintains the same sign for long enough, it will become saturated at a low or a high output level, for a negative and a positive difference, respectively. This regime can be used in feedbacks requiring time integration (integral feedbacks), such as the one proposed to operate in bacterial chemotaxis [62].

Regime 2: kinase saturated, phosphatase unsaturated : signal-transducing

Of the two new regimes that we characterize in this study, the one with a saturated kinase and unsaturated phosphatase (Figure 2B) is of particular interest. We refer to this regime as signal-transducing because, as discussed below, it is ideal for transmitting noisy time-varying signals. Equation 3 for this regime becomes

$$\frac{d\overline{A}}{dt} = k_1\overline{E_1} - k_2\frac{\overline{E_2}}{K_2 + \overline{E_2}}\overline{A},$$

which is linear in $\overline{A}$. This has several interesting implications. In particular, it implies that for slow inputs (relative to the cut-off frequency $k_2\overline{E_2}/(K_2 + \overline{E_2})$) the output $\overline{A}$ will simply be a scaled copy of the input. While the latter holds for slow inputs, quickly varying inputs (noise) are filtered out making this regime ideal for the transmission of signals. Furthermore, the fact that this cycle is a linear system implies that pathways (or part of pathways) built of cycles in this regime become highly tractable mathematically since all the well-developed signals and systems techniques would apply to them. Available biochemical data and in vivo measurements argue in favor of this regime to be present in cell signaling cascades (see Discussion).

Regime 3: kinase unsaturated, phosphatase saturated : threshold-hyperbolic

The second new regime has an unsaturated kinase and a saturated phosphatase, and Equation 3 becomes

$$\frac{d\overline{A}}{dt} = k_1\frac{\overline{E_1}(\overline{S} - \overline{A})}{K_1 + \overline{E_1}} - k_2\overline{E_2}.$$

Its steady state output is zero for inputs below a threshold and then increases hyperbolically with increasing steady-state inputs.

Regime 4: both kinase and phosphatase unsaturated : hyperbolic

This regime was also first identified in [27] and exhibits a hyperbolic steady state response. Equation 3 becomes

$$\frac{d\overline{A}}{dt} = k_1 \frac{\overline{E_1}(\overline{S} - \overline{A})}{K_1 + \overline{E_1}} - k_2 \frac{\overline{E_2}\,\overline{A}}{K_2 + \overline{E_2} + \overline{A}}$$

for this regime.
