# Supplementary material for: Operating Regimes of Signaling Cycles: Statics, Dynamics, and Noise Filtering
Source: PLoS Comput Biol. 2007 Dec 21;3(12):e246. doi: 10.1371/journal.pcbi.0030246 (PMC2230677; doi:10.1371/journal.pcbi.0030246)
Supplement: Text S3 — (152 KB PDF) [file pcbi.0030246.sd003.pdf]

Text S3

Steady State

The output of the cycle is the amount of free active protein and may be found from the amount of active protein $\overline{A}$ and of active complex C_2, since $A = \overline{A} - C_2$. Analytic approximations to the steady state response of the signaling cycle may then be obtained by finding expressions for $\overline{A}$ and C_2. The former can be found by setting the left hand side of Equation 3 to zero and solving for $\overline{A}$, while the latter is taken to be $\frac{\overline{E_2}\,\overline{A}}{K_2+\overline{E_2}+\overline{A}}$ as discussed in the appendix above.

So doing for the four signaling regimes results in analytic expressions for their steady state responses. The case of the ultrasensitive regime, however, involves a slightly different method.

Regime 1: ultrasensitive

Setting Equation 3 to zero for this regime results in $k_1\overline{E_1} = k_2\overline{E_2}$, and since for this regime $C_2 \approx E_2$ then this indicates that $C_2 \approx \frac{k_1}{k_2}\overline{E_1}$. Numerical simulation indicates that as long as $\frac{k_1}{k_2}\overline{E_1} \leq \overline{E_2}$ the previous relation is accurate and furthermore that the switch output is zero. As the input increases beyond this point, C_2 quickly increases to its maximal value $\overline{E_2}$ (i.e., the phosphatase becomes fully saturated, while the level of free inactive protein decreases to zero and the inactive complex $C_1 \approx \overline{E_2}\frac{k_2}{k_1}$. Together, these observations imply that for $\overline{E_1} \leq \frac{k_2}{k_1}\overline{E_2}$ the output of the cycle is zero and $\overline{A} \approx C_2 \approx \frac{k_1}{k_2}\overline{E_1}$, and that for inputs above this level $\overline{A}$ quickly saturates at $\overline{S} - \frac{k_2}{k_1}\overline{E_2}$, and the output level is given by $A = \overline{S} - \left(1 + \frac{k_2}{k_1}\right)\overline{E_2}$. This implies that no matter how high the input is, the output of the ultrasensitive cycle will never equal the total amount of substrate protein unless there is no phosphatase.

Regime 2: signal-transducing

Setting Equation 3 to zero for this regime results in $k_1\overline{E_1} - k_2\frac{\overline{E_2}}{K_2+\overline{E_2}}\overline{A} = 0$, so $\overline{A} = \frac{k_1}{k_2}\frac{K_2+\overline{E_2}}{\overline{E_2}}\overline{E_1}$. At the same time $C_2 \approx \frac{\overline{E_2}}{K_2+\overline{E_2}}\overline{A}$ so that $A = \frac{k_1}{k_2}\left(\frac{K_2+\overline{E_2}}{\overline{E_2}} - 1\right)\overline{E_1}$. This linear relationship between the output and the input can not hold for high inputs because the output must be less than the total amount of substrate. We therefore expect the output to saturate when there is not free inactive protein, i.e., when $\overline{A} + C_1 \approx \overline{S}$. Since $C_1 \approx \overline{E_1}$ in this regime, the previous expression implies that the switch will saturate when $\overline{E_1} \approx \frac{\overline{S}}{1+\frac{k_1}{\omega_2}}$, where $\omega_2 = \frac{k_2\overline{E_2}}{K_2+\overline{E_2}}$. Evaluating the output at this input level yields the saturation value of the switch in this regime: $A = \left(1 - \frac{\omega_2}{k_2}\right)\left(\frac{\frac{k_1}{\omega_2}}{1+\frac{k_1}{\omega_2}}\right)\overline{S}$.

Regime 3: threshold-hyperbolic

Setting Equation 3 to zero for this regime results in $\omega_1(\overline{S} - \overline{A}) - k_2\overline{E_2} = 0$, where $\omega_1 = k_1\frac{\overline{E_1}}{K_1+\overline{E_1}}$. This implies that $\overline{A} \approx \overline{S} - \frac{k_2}{\omega_1}\overline{E_2}$ and since $C_2 \approx \overline{E_2}$, that $A \approx \overline{S} - (1+\frac{k_2}{\omega_1})\overline{E_2}$. This approximation is not expected to hold at low inputs, where it blows up. Instead, at low inputs the free active protein is expected to be zero and $\overline{A} \approx C_2 \approx \frac{\omega_1}{k_2}(\overline{S} - \overline{A})$ from the first expression in this

subsection. Solving for $\overline{A}$ gives $\overline{A} \approx \frac{\omega_1}{k_2+\omega_1}\overline{S} \approx A$ for low inputs. This expression is expected to break as the input level reaches a level $\overline{E_1}^*$ where the expression equals $\overline{E_2}$. Above that input the first expression for $\overline{A}$ is expected to hold. Therefore, for inputs below $\overline{E_1}^*$ the output is approximately zero, and then increases hyperbolically as $A \approx \overline{S} - (1 + \frac{k_2}{\omega_1})\overline{E_2}$.

Regime 4: hyperbolic

Setting Equation 3 to zero for this regime results in $\omega_1(\overline{S} - \overline{A}) - \omega_2\overline{A} = 0$, where ω_1 and ω_2 are as defined above. Therefore $\overline{A} \approx \frac{\omega_1}{\omega_1+\omega_2}\overline{S}$ and since $C_2 \approx \frac{\omega_2}{k_2}\overline{A}$ then $A = \left(1 - \frac{\omega_2}{k_2}\right)\frac{\omega_1}{\omega_1+\omega_2}\overline{S}$. The saturation level of this regime is obtained by evaluating the previous expression in the limit as $\overline{E_1}$ becomes infinite.
