# Supplementary material for: Operating Regimes of Signaling Cycles: Statics, Dynamics, and Noise Filtering
Source: PLoS Comput Biol. 2007 Dec 21;3(12):e246. doi: 10.1371/journal.pcbi.0030246 (PMC2230677; doi:10.1371/journal.pcbi.0030246)
Supplement: Text S4 — (150 KB PDF) [file pcbi.0030246.sd004.pdf]

Text S4

Dynamics

To find approximate analytic expressions for the response of the system to inputs of the form $\overline{E_1} = E_0(1 + a\sin\omega t)$, we use small signal analysis. This method consists of linearizing the system about its steady state level, and further assuming that the input deviates from its steady state level by small amounts. Any results thus obtained are expected to be valid for small $E_0 a$, although numerically we have observed that the results so obtained describe the system better than they might have the right to when $E_0 a$ is not small. The method works as follows: First let the function $f(\overline{A}, \overline{E_1})$ (or just f for simplicity) denote the rate of change of $\overline{A}$ as described by Equation 3 (i.e., $\frac{d\overline{A}}{dt} = f(\overline{A}, \overline{E_1})$), and let $\overline{A}_{ss}$ be the steady state level of $\overline{A}$ when the input is constant and equal to E_0, so that $f(\overline{A}_{ss}, E_0) = 0$. Then define the deviations from steady state levels $\delta\overline{A} = \overline{A} - \overline{A}_{ss}$ and $\delta\overline{E_1} = \overline{E_1} - E_0 = E_0 a \sin\omega t$. Assuming the deviations are always small and Taylor expanding $f(\overline{A}, \overline{E_1})$ about the steady state levels then yields

$$\frac{d\delta\overline{A}}{dt} = g\delta\overline{E_1} - \omega_c\delta\overline{A}, \tag{9}$$

where $g = \frac{\partial f}{\partial \overline{E_1}}\big|_{(\overline{A}_{ss}, E_0)}$ is referred to as the gain and $\omega_c = \frac{\partial f}{\partial \overline{A}}\big|_{(\overline{A}_{ss}, E_0)}$ as the cut-off frequency. This equation is linear and may be solved for arbitrary inputs $\delta\overline{E_1}$ by one of the many useful techniques to work with linear differential equation (i.e., by Laplace transforms). In particular, when $\delta\overline{E_1} = aE_0 \sin\omega t$ and the initial condition is zero

$$\delta\overline{A} = aE_0 \frac{g}{\sqrt{\omega^2 + \omega_c^2}} \cos\left(\omega t + \tan^{-1}\left(\frac{-\omega_c}{\omega}\right)\right) + aE_0 g\omega e^{-\omega_c t},$$

where $\tan^{-1}$ denotes the inverse tangent. Here, we are only interested in twice the amplitude of the steady state oscillations in $\overline{A}$, from maximum to minima. These are evidently given by Equation 4, such that for frequencies smaller than the cut-off ω_c the oscillations are proportional to $\frac{g}{\omega_c}$ and oscillations for frequencies larger than ω_c decay as $1/\omega$.

Because the output of the system is $A = \overline{A} - C_2$, we need to translate these oscillations in $\overline{A}$ to oscillations in A. In the ultrasensitive and threshold-hyperbolic regimes, $C_2 \approx \overline{E_2}$ so the oscillations in A equal those in $\overline{A}$. In the hyperbolic and signal-transducing regimes, $C_2 \approx \frac{\omega_2}{k_2}\overline{A}$, so the amplitude of the oscillations in A is that amplitude of the oscillations in $\overline{A}$ multiplied by a factor of $1 - \frac{\omega_2}{k_2}$.

Regime 1: ultrasensitive

For the ultrasensitive regime we do not need to use the method above. This regime needs to be fine-tuned to transmit signals because, as evidenced by its steady state response curve, is only responsive to changes in the input close to its inflection point, at $\overline{E_1} = \frac{k_2}{k_1}\overline{E_2}$. Choosing E_0 at this level results in the cycle equation becoming $\frac{d\overline{A}}{dt} = k_1 a E_0 \sin\omega t$, which is identical to Equation 9 with a gain of k_1 and cut-off frequency of zero. The previous equation does not hold for small enough frequencies; instead at some effective cut-off frequency the oscillations

will cover the full range of values that the ultrasensitive cycle may take. That is, the effective cut-off frequency satisfies $2E_0 a \frac{k_1}{\omega_c} = \overline{S} - \left(1 + \frac{k_2}{k_1}\right)\overline{E_2}$, where the right hand side is the saturation level of the cycle. Solving for ω_c in this expression yields the cut-off frequency in Table 3. The ultrasensitive regime is the only one that achieved oscillations that cover its full steady state response range, and where the (effective) cut-off frequency depends on the input amplitude a.

Regime 2: signal-transducing

Because Equation 3 for this regime is already linear in $\overline{A}$ and in $\overline{E_1}$, it already has the same form as Equation 9 with $g = k_1$ and $\omega_c = \omega_2$. Multiplying the gain by $1 - \frac{\omega_2}{k_2}$ to translate to oscillations in $\overline{A}$ gives the result in Table 3.

Regime 3: threshold-hyperbolic

Applying the method described above results in the expressions in Table 3 (These results are not expected to hold when the steady state input E_0 is below the regime's threshold and the output is zero). For simplicity though, we let $\omega_0 = \omega_1\big|_{(\overline{E_1}=E_0)} = \frac{k_1 E_0}{K_1 + E_0}$, which turns out to be ω_c for this regime.

Regime 4: hyperbolic

Applying the method described above results in the expressions in Table 3, where the cut-off turns out to be $\omega_c = \omega_0 + \omega_2$.
