# Supplementary material for: Operating Regimes of Signaling Cycles: Statics, Dynamics, and Noise Filtering
Source: PLoS Comput Biol. 2007 Dec 21;3(12):e246. doi: 10.1371/journal.pcbi.0030246 (PMC2230677; doi:10.1371/journal.pcbi.0030246)
Supplement: Text S5 — (73 KB PDF) [file pcbi.0030246.sd005.pdf]

Text S5

Quantifying the Quality of the Four Regime Approximations

Taking extreme values of the kinase and phosphatase MM constants allows us to obtain the four signaling regimes previously discussed. However, the results obtained from these approximations apply reasonably well to a wide range of MM constants, and not only at the extreme. The quality of the approximation does increase, however, as the MM constants become more extreme. To demonstrate this we numerically solved for the steady-state characteristic of Equation 3 for a wide range of kinase and phosphatase MM constants and compared them to the characteristics of each of the four regimes. For each set of K_1 and K_2 values, we set the left hand side of Equation 3 to zero and solve for $\overline{A}$ and then subtract $\frac{\overline{E_2}\,\overline{A}}{K_2+\overline{E_2}+\overline{A}}$ as discussed in the section above to obtain A. We do so for a range of total kinase values K_t and for each, we compute the difference from the steady-state of each of the four regime steady states. We finally square these differences and compute their mean resulting in the mean squared error for each regime. In Figure 3 we show what we refer to a the relative error, the square root of the squared error normalized by the total substrate S_t. This figure again shows that the regime approximations are each approximately valid over a large part of a quadrant, covering almost the full K_1 versus K_2 space when combined.
