# Supplementary material for: Operating Regimes of Signaling Cycles: Statics, Dynamics, and Noise Filtering
Source: PLoS Comput Biol. 2007 Dec 21;3(12):e246. doi: 10.1371/journal.pcbi.0030246 (PMC2230677; doi:10.1371/journal.pcbi.0030246)
Supplement: Text S6 — (72 KB PDF) [file pcbi.0030246.sd006.pdf]

Text S6

Low-pass filtering

Figure S1 summarizes the low-pass filtering behavior for the four regimes. It shows O (color coded) versus a and ω (i.e., a horizontal cut through this plot would simply be an O versus ω curve such as those shown in Fig. 5). Some salient features are evident in this figure. (i) All regimes act as low-pass filters. (ii) Although Equation 4 is obtained using a small signal approximation and is expected to hold for small a, it provides a good guide for describing O for all values of a. Perhaps the biggest discrepancy is the fact that O does not increase linearly with a but saturates (see Fig. S1A,C,D). The signal-transducing regime, however, does seem to have a response that increases linearly with a so for a given frequency an input with twice the amplitude of another will result in twice the output O. (iii) Finally, for all regimes except the ultrasensitive regime (Fig. S1D) the response starts decreasing at about the same frequency, independent of a, where as for the ultrasensitive cycle smaller a results in smaller cut-off frequency.

The ultrasensitive regime

The ultrasensitive cycle is the only cycle that oscillates between a level close to its saturation value and zero for a wide range of inputs (red region of Fig. S1D). The cutoff frequency of this cycle is a function of both the total substrate protein of the cycle, and of the input's amplitude, a unique property of this cycle. For all other cycles, the cutoff frequency is independent of total substrate and of the input parameters.

The signal-transducing regime

The cutoff frequency depends only on the phosphatase level and phosphatase parameters, but the gain depends on both phosphatase and kinase parameters. Thus the cutoff frequency can be tuned by changing phosphatase parameters or level, and the gain can be independently adjusted by changing the catalytic rate of the kinase via evolution.

The threshold hyperbolic regime

The cutoff frequency depends only on the average kinase level and kinase parameters. Thus the cutoff frequency may be tuned by changing the kinase level and/or parameters, and the gain may be tuned independently by adjusting phosphatase levels or parameters.

The hyperbolic regime

This cycle has a cutoff frequency that depends on both the kinase and the phosphatase. Increasing either one increases the cutoff frequency. The gain also depends on both the kinase and the phosphatase, so adjusting their levels will modify both the gain and the cutoff frequency of the switch.
